# The role of klotho in chronic kidney disease

**DOI:** 10.1186/s12882-018-1094-z

**Published:** 2018-10-22

**Authors:** Di Zou, Wen Wu, Yan He, Sichao Ma, Ji Gao

**Affiliations:** 1 Department of Nephrology, The First Affiliated Hospital to Changchun University of Chinese Medicine, Changchun, Jilin, 130000 China; 2 Department of Medical Ultrasonics, PLA Army General Hospital, Beijing, 100700 China; 3 Department of Urology, China-Japan Union Hospital, Jilin University, Changchun, Jilin, 130000 China

**Keywords:** Chronic kidney disease, Extrarenal complications, Klotho functions, Pathogenic mechanism, Potential applications

## Abstract

Chronic kidney disease (CKD) is an inherently systemic disease that refers to a long-term loss of kidney function. The progression of CKD has repercussions for other organs, leading to many kinds of extrarenal complications. Intensive studies are now being undertaken to reveal the risk factors and pathophysiological mechanism of this disease. During the past 20 years, increasing evidence from clinical and basic studies has indicated that klotho, which was initially known as an anti-aging gene and is mainly expressed in the kidney, is significantly correlated with the development and progression of CKD and its complications. Here, we discuss in detail the role and pathophysiological implications of klotho in ion disorders, the inflammation response, vascular calcification, mineral bone disorders, and renal fibrosis in CKD. Based on the pathogenic mechanism of klotho deficiency and klotho decline in urine early in CKD stage 2 and even earlier in CKD stage 1, it is not difficult to understand that soluble klotho can serve as an early and sensitive marker of CKD. Moreover, the prevention of klotho decline by several mechanisms can attenuate renal injuries, retard CKD progression, ameliorate extrarenal complications, and improve renal function. In this review, we focus on the functions and pathophysiological implications of klotho in CKD and its extrarenal complications as well as its potential applications as a diagnostic and/or prognostic biomarker for CKD and as a novel treatment strategy to improve and decrease the burden of comorbidity in CKD.

## Background

Chronic kidney disease (CKD) is a progressive systemic disease that irreversibly alters the function and structure of the kidney, over months or years. CKD progression has repercussions for other organs, exerting multiple negative systemic effects on numerous organs, including those of the cardiovascular system, leading to cardiovascular diseases, which increase the risk of mortality [1]. In the past 3 decades, Intensive studies in animals and humans have been performed to reveal the risk factors and pathophysiological mechanism of this disease. These studies have established that the original disease process causes an initial loss of nephron unit; then, renal diseases progress to renal failure as a consequence of functional adaptations intervening in the kidney, leading to injury in other organs. Moreover, many factors are involved in this process, including a variety of cytokines, growth factors and vasoactive substances [2, 3]. Recently, more evidences has suggested that the development and progression of CKD are significantly associated with a decline in klotho, which was initially described as an anti-aging gene [4–7].

The klotho gene is mainly expressed in the cell surface membrane of proximal and distal renal tubules [8–12]. Uder normal physiological conditions, the kidney is a major regulator that helps maintain klotho levels [6, 13, 14]. However, in individuals and in animal models with CKD, klotho levels decline and are accompanied by renal insufficiency [7, 15]. Experimentally, klotho-deficient mice and CKD subjects have similar phenotypes, suggesting that klotho is tightly correlated with the pathogenic mechanism of CKD [4, 7]. Furthermore, further evidence has shown that klotho is not only an early biomarker of CKD, but also a potential therapeutic target for CKD [14, 16, 17]. Thus, based on the relationship between klotho and CKD, we here systematically review the functions, physiopathological characteristics, and potential applications of klotho in the related signs and complications of CKD.

## Main text

### The klotho family and structure

Klotho family members include α-, β-, and γ-klotho genes based on their predicted primary sequences [18, 19]. β- and γ-klotho were discovered based on their homology with α-klotho, and they all share a single-pass transmembrane protein [20, 21]. β-Klotho is predominantly expressed in the liver but is also found in the kidney, gut, and spleen and mediates the activity of members of the fibroblast growth factor (FGF) family, such as FGF-19 and -21 [18, 22]. γ-Klotho is expressed in the kidney and skin and has undefined functions [18, 21]. In this review, we only focus on α-klotho; the term klotho in the following paragraphs refers to α-klotho.

**Fig. 1:**
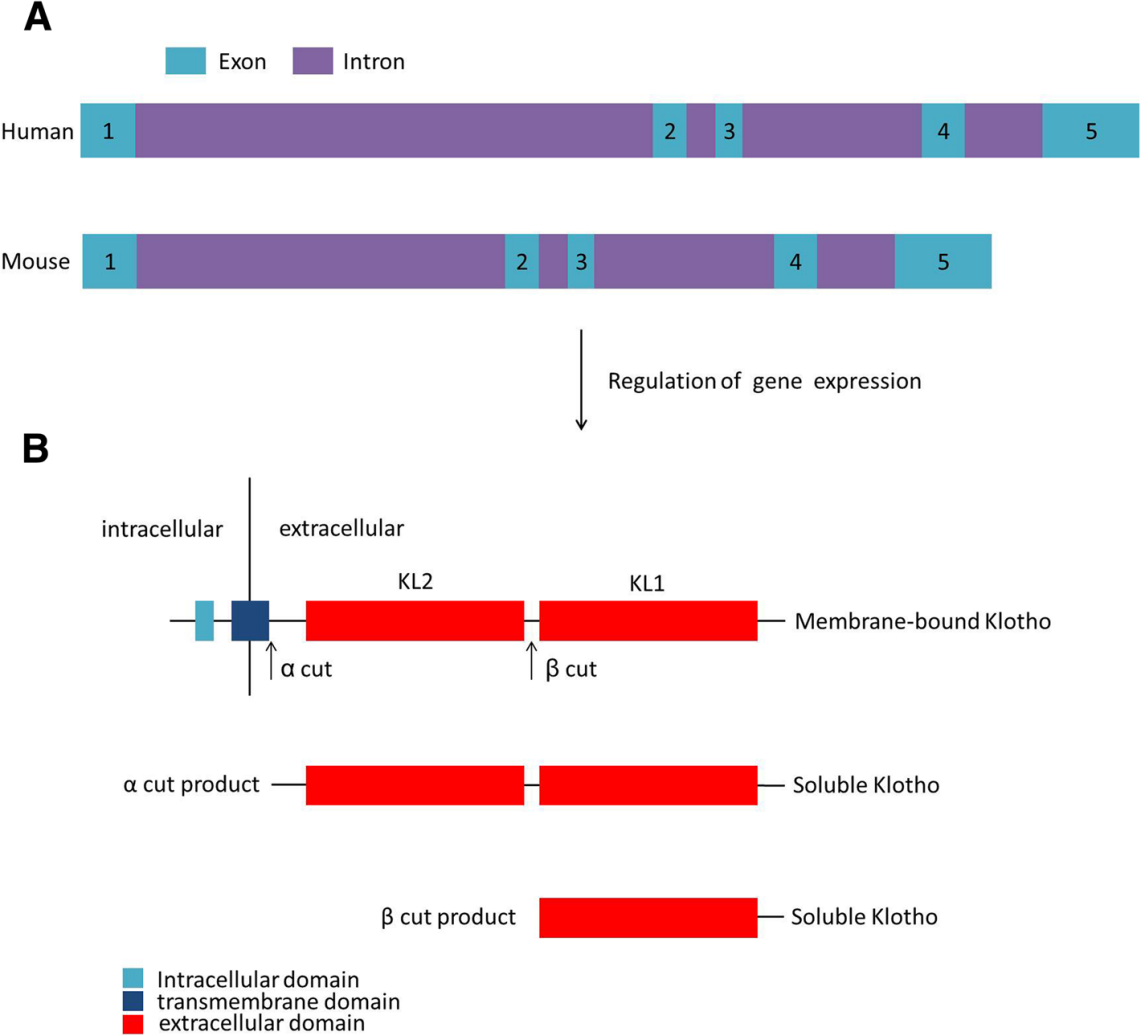
The structure of the klotho gene and protein. **a** The structure of the klotho genes from humans and mice. **b** The structure of the klotho protein. Soluble klotho proteins are generated by full-length transcript splicing at the indicated α and β cut sites, comprising the entire extracellular domain, or at the single Kl1 or Kl2 domains

α-Klotho is composed of five exons that correspond 1,012 amino acids in the human protein and 1,014 amino acids in the mouse protein (Fig. 1a) [23]. The protein consists of a large extracellular domain, including 980 N-terminal residues followed by a 21-amino-acid transmembrane domain and a small domain of 11 residues corresponding to the intracellular C-terminus [10, 24]. The extracellular domain of membrane klotho consists of two repeat sequences of 440 amino acids termed Kl1 and Kl2, which are generated by full-length transcript splicing and can be cleaved by the metalloproteinases ADAM-10 and ADAM-17 and released into circulation as soluble klotho (cleaved klotho) (Fig. 1b) [10, 25–27]. In addition, an alternatively spliced klotho mRNA transcript has been hypothesized to code for a secreted klotho protein, which would equate to the Kl1 domain, but this putative protein has not been identified and has not been detected in human serum thus far; it has been observed only in in vitro systems [24, 28, 29]. Furthermore, a recent study showed that this alternative klotho mRNA was degraded by nonsense-mediated mRNA decay (NMD), finally resulting in no active protein translation [30]. Soluble klotho is the main functional form in the circulation [1, 31] and is detected in the blood, urine, and cerebrospinal fluid [31–34], exerting its function by acting as a hormone. Additionally, another functional form of klotho occurs, termed membrane-bound klotho, which is mainly involved in FGF receptor signalling.

### The function and pathophysiological implications of klotho in CKD

#### Co-receptor of FGF23

FGF23 belongs to the FGF family. Many studies have shown that FGF23 not only increases the urinary excretion of phosphate but also indirectly suppresses intestinal phosphate absorption by down-regulating the production of 1α,25-dihydroxyvitamin D3 (1,25(OH)_2_D) [12, 35–37]. FGF23 transduces signals by binding to its receptors to phosphorylate downstream signalling molecules [38]. There are four different FGF receptors (FGFRs), FGFR1-4; these proteins are tyrosine kinase receptors and have high or low affinity for FGFs. Because of their lack of a heparan sulfate-binding domain, FGF23 requires full-length klotho to convert the canonical FGFR into a specific high-affinity receptor to function in target tissues [39, 40]. Recently, research has shown that in the complex consisting of the shed extracellular domain of klotho, the FGFR1c ligand-binding domain, and FGF23, klotho simultaneously tethers FGFR1c by its D3 domain and FGF23 by its C-terminal tail, thus resulting in FGF23-FGFR1c proximity and conferring stability [41]. Thus, klotho is an essential co-receptor for the binding of FGF23 to its receptors.

In proximal renal tubules, blood-borne FGF23 binds to FGFR-klotho complexes and directly activates extracellular signal-regulated kinase (ERK)1/2 and serum/glucocorticoid-regulated kinase (SGK)-1 signals. Subsequently, SGK-1 phosphorylates the Na^+^/H^+^ exchange regulatory cofactor (NHERF)-1 to down-regulate membrane expression of the key sodium phosphate cotransporter NaPi-2a, thus leading to an increase in urinary phosphate excretion [12, 42–44]. Loss of membrane-bound klotho expression limits FGF23-stimulated signal transduction through FGFR-klotho complexes. It has been shown that a specific deletion of klotho in proximal renal tubules was unable to increase renal phosphate excretion in vivo [45], suggesting that the effect of FGF23 on phosphate excretion is limited by proximal tubular klotho deficiency. Furthermore, FGF23 suppresses renal 1α-hydroxylase expression, which is the key enzyme responsible for 1,25(OH)_2_D production, by a klotho-dependent signalling mechanism in proximal renal tubules [46–48]. In addition, soluble klotho directly regulates phosphorus excretion in the kidney and participates in systemic mineral homeostasis by regulating 1α-hydroxylase activity and parathyroid hormone (PTH) and FGF23 secretion [49, 50]. These results suggest that klotho deficiency limits its regulation of FGF23 production and hyperphosphataemia remains the principal regulator of FGF23 secretion in CKD [51]. The effect of FGF23 on both phosphate and 1,25(OH)_2_D is involved in FGFR1, FGFR3, and FGFR4, especially FGFR1 [52, 53]. In addition, it has been identified that as with the mineral parameters, FGF23 and phosphate are increased, while klotho and 1,25(OH)_2_D are decreased in CKD, especially in early stages, except serum phosphate [54–56]. These changes in mineral parameters play a central role in the pathophysiology of CKD [19]. Notably, each disturbance in the mineral parameters can be pathogenic alone or can drive and exaggerate the disturbance of the other parameters [18, 19].

It was previously reported that soluble klotho is a regulator of the epithelial calcium channel transient receptor potential vannilloid-5 (TRPV5), a glycoprotein that is essential for the entry of calcium into calcium-transporting renal epithelial cells [57]. TRPV5 regulation by klotho is thought to operate as follows: soluble klotho specifically hydrolyses sugar residues from the glycan chains on TRPV5, which in turn stabilizes TRPV5 in the membrane through interaction of the sugar residues with extracellular galectin [57, 58]. However, the cellular secretion process of klotho is unclear. Recently, a decrease in renal calcium reabsorption and a renal membrane abundance of TRPV5 were observed in klotho-knockout mice, similar to the finding in FGF23-knockout mice, but klotho neither co-localizes with TRPV5 nor is regulated by FGF23. Rather, Andrukhova O et al. supported the notion that the apical membrane abundance of TRPV5 in renal distal tubules and renal calcium reabsorption are regulated by FGF23 through binding the FGFR-klotho complexes [59]. Based on these results, it can be propsed that FGF23 functions by binding to FGFR-klotho complexes, thus directly modulating calcium reabsorption in distal renal tubules. In contrast, hypocalcaemia (calcium deficiency) reduces the circulating concentrations of FGF23 [60]. This decrease in FGF23 might be a response that avoids a subsequent reduction in calcitriol, which could exacerbate hypocalcaemia. Additionally, Andrukhova O et al. found that FGF23 directly regulates sodium reabsorption in distal renal tubules by a signalling mechanism involving the FGFR-klotho complexes and the activation of ERK1/2, SGK1, and with-no-lysine kinase 4 (WNK4) signal cascades, suggesting that FGF23 is also a key regulator of renal sodium reabsorption and plasma volume [61]. This may explain the association of FGF23 with cardiovascular risk in CKD patients. Due to calcium and sodium disregulation in renal diseases [62], the novel link between FGF23 and the metabolism of these ions may have major pathophysiological implications in CKD [12].

Noticeably, membrane receptors of soluble klotho have not previously been identified. A recent study found that α2-3-sialyllactose, which is present in the glycan of monosialogangliosides, is a receptor of soluble klotho. Soluble klotho binds to ganglioside-enriched lipid rafts to regulate PI3K signalling [63]. Furthermore, another study identified the key protein residues in the Kl1 domain that are likely involved in binding to α2-3-sialyllactose, which down-regulates TRPC6 channels and protects against stress-induced cardiac hypertrophy [64]. These results provide new insight that targeting sialic acids may be a general mechanism underlying the pleiotropic actions of soluble klotho.

#### Anti-inflammation

Inflammation is multifactorial in CKD, and this disease is considered a prototypical example of inflammatory disease and premature ageing [65, 66]. There are many proinflammatory factors increased gradually in CKD as renal function fails, including interleukin (IL)-6, serum fetuin-A, and tumour necrosis factor (TNF) [66, 67]. Nuclear factor κB (NF-κB) controls many cellular processes, such as antiapoptotic responses, oxidative stress, and especially, inflammatory responses [68]. In normal situations, NF-κB is located in the cytoplasm in an inactive form, linked to its inhibitory proteins,termed inhibitory κB (IκB). In response to various stimuli, such as TNF, two serine residues at positions 32 and 36 in the N-terminal region of IκB are phosphorylated. This phosphorylation induces IκB ubiquitination by the E3-IκB ubiquitin ligase complex, causing its degradation by the 26S proteosome, thus leading to NF-κB translocation to the nucleus and the direct activation of downstream gene transcription [23, 68]. Greater NF-κB activity increases the expression of proinflammatory mediators, such as cytokines and adhesion molecules. Several studies have shown that NF-κB plays a pivotal role in the progression of chronic renal inflammation, whereby the inhibition of NF-κB reduces the levels of several proinflammatory cytokines and renal injury [69–71].

One study has shown that there is a bidirectional relationship between klotho and NF-κB [23]. On the one hand, klotho expression is down-regulated by an NF-κB–dependent mechanism. Reduced klotho in the blood and urine has been observed in human CKD [72, 73]. In a nephrotoxic acute kidney injury (AKI) mouse model, klotho expression was also reduced, and blockage of TNF-related weak inducer of apoptosis (TWEAK), which is a member of the TNF superfamily, was able to revert kidney klotho levels and preserve renal function. Moreover, the inhibition of NF-κB prevents TWEAK-mediated decreases in klotho levels [74]. Thus, proinflammatory cytokines, such as TWEAK, negatively regulate the expression of klotho through an NF-κB–dependent mechanism, and NF-κB is a key contributor to the regulation of klotho expression [23].

On the other hand, klotho is an anti-inflammatory modulator that negatively regulated NF-κB, consequently leading to a decrease in proinflammatory gene transduction. It has been reported that TNF increases vascular cell adhesion protein 1 (VCAM-1) and intercellular adhesion molecule 1 (ICAM-1) expressions in endothelial cells, while klotho can suppress TNF-induced increases in ICAM-1 and VCAM-1 expression by attenuating NF-κB activity [75]. Furthermore, in klotho-mutated mice, the exogenous addition of soluble klotho or the overexpression of membranous klotho in tissue culture suppresses NF-κB activation and NF-κB–mediated inflammatory cytokines via a mechanism that involves the phosphorylation of serine(536) in the transactivation domain of RelA [76]. Similarly, an excess of klotho inhibits the PDLIM2/NF-κB pathway to decrease the production of TNF-α, IL-6, and IL-12, and to ameliorate cyclosporine A-induced nephropathy in vivo and in vitro [77]. In addition, klotho can suppress NADPH oxidase 2 (Nox2) protein expression and attenuate oxidative stress in rat aortic smooth muscle cells and can also suppress retinoic acid-inducible gene-I (RIG-I)-mediated inflammation [78, 79]. Thus, klotho may act as an anti-inflammatory modulator in the kidney.

#### Protection against vascular calcification and mineral bone disorder

Vascular calcification (VC) appears early in the course of CKD but becomes much more prevalent as kidney function deteriorates, creating a strong risk of cardiovascular mortality and morbidity in patients with CKD and ESRD [80, 81]. VC can be classified based on the vascular site of abnormal mineral deposition, including intimal calcification, medial calcification, and valvular calcification, which are all highly prevalent in the CKD population [82, 83]. It is now clear that VC is a cell-regulated pathological process that involves many inhibitors and inducers [5]. Under normal conditions, several inhibitors protect against VC by calcium and phosphate supersaturation, such as pyrophosphate, matrix Gla protein, and fetuin-A [84–88]. In the CKD population, the total function between inhibitors and inducers is unbalance, leading to the occurrence of VC in the vessel walls and valves. There are many inducers of VC in CKD, including hypercalcaemia, inflammatory cytokines, and especially, phosphate [88, 89]. Clinical evidence showed that the upregulation of serum phosphate is one of many risk factors for VC in the CKD population [90, 91]. Moreover, a growing amount of experimental research has revealed the mechanism of phosphate-induced VC, showing that PiT-1 which is phosphate cotransporter in vascular smooth muscle cells (SMCs), is involved in pathogenesis and promotes VC by induction of SMCs osteochondrogenic transformation and apoptosis and by regulation of extracellular vesicles release and stability [88, 92–94]. These results suggest that elevated phosphate is a main inducer of VC.

The expression level of klotho decreases in patients with CKD and animal models, and is accompanied by renal disorders [7, 15]. It has been reported that klotho deficiency causes high circulating levels of Phosphate and VC occurrence in mice with CKD. Conversely, overexpression of klotho can enhance phosphaturia, improve renal function, and produce much less calcification in vivo as well as suppress the sodium-dependent uptake of Phosphate and Phosphate-induced calcification of rat vascular SMCs [7]. Zhang et al. reported that cleaved klotho protein attenuates the Phosphate-induced human bone marrow mesenchymal stem cells differentiation into osteoblast-like cells in vitro via inactivation of the FGFR1/ERK signalling pathway [95]. In addition, the up-regulation of klotho expression by the inhibition of rapamycin signalling also ameliorates VC and protects against vascular disease in CKD [96, 97]. Another study showed that Intermedin 1-53 attenuates VC in rats with CKD by up-regulating membrane-bound klotho expression in the vessel wall [98]. Recent studies have confirmed that the stable delivery of soluble klotho can reduce chronic hyperphosphataemia and VC in vitro and in vivo [99], and activating peroxisome proliferator-activated receptor γ enhanced the expression of klotho to inhibit Phosphate-induced VC in vascular SMCs [100]. These results suggested that klotho deficiency is closely associated with hyperphosphataemia and VC and that enhancing klotho activity plays a protective role in hyperphosphataemia and VC in CKD.

CKD-mineral bone disorder (MBD) is a newly termed systemic disorder that begins early in stage 2 of CKD and is characterized by abnormal serum biochemistries including hyperphosphataemia and hypercalacemia, bone disorders, and VC [88, 101]. The causes of VC and cardiovascular mortality associated with CKD are partly attributed to CKD-MBD [51, 102, 103]. Recent studies demonstrate that factors that are involved in renal injury and repair and that are released into the circulation contribute to the pathogenesis of CKD-MBD [51]; such factors include the Wnt signal inhibitors, Dickkopf 1 [104, 105] and sclerostin [106, 107], as well as activin A and ActRIIA [108, 109]. The pathogenic mechanisms of the components of CKD-MBD include VC, loss of renal klotho, hyperphosphataemia, osteodystrophy, vitamin D deficiency, increased FGF23, cardiovascular disease, and hyperparathyroidism [51]. In this review, we mainly focus on the aspects related to klotho. As described previously, the expression of klotho is significantly decreased in CKD. It has been reported that this decrease in klotho is partly related to activin and ActRIIA signalling. Furthermore, the activation of ActRIIA signalling by using a ligand trap for the receptor significantly stimulates klotho levels [108]. The resulting reduction in klotho limits its regulation of FGF23 production and leaves hyperphosphataemia as the principal regulator of FGF23 secretion in CKD [51]. Recently, researchers have identified that klotho loss is a key event in the renal and bone injuries in CKD-MBD mice, and endogenous klotho restoration by histone deacetylase inhibition attenuates CKD-associated bone complications in a mouse model of CKD-MBD [110]. Similarly, rhein-regulated klotho expression by promoter hypermethylation protects against renal and bone injuries in mice with CKD. When klotho is knocked down by RNA interference, the renal protective effects of rhein are largely abolished [111]. These data suggest that klotho deficiency is closely associated with the development of CKD-MBD and that klotho restoration is beneficial to the improvement of VC and CKD-MBD.

#### Amelioration of renal fibrosis

The final common pathological manifestation of many instances of CKD is renal fibrosis. Renal fibrosis represents the unsuccessful wound healing of kidney tissue after chronic, sustained injury and is characterized by glomerulosclerosis, tubular atrophy, and interstitial fibrosis [112]. The progression of CKD is evidenced by a loss of renal cells and their replacement by extracellular matrix (ECM) in the glomeruli and interstitium [66, 113]. The pathogeneses of glomerulosclerosis and tubulointerstitial fibrosis are extremely similar [113]. In essence, renal injury results in an inflammatory cascade involving macrophage activation and T-cells recruitment, triggering an immune response and causing interstitial nephritis. Then, several cell types including macrophages, T-cells, and tubular epithelial cells respond to this inflammatory process to produce profibrotic mediators, such as transforming growth factor β (TGF-β). Under the influence of profibrotic cytokines, injured tubular epithelial cells dedifferentiate and lose their polarity and transporter function, reorganize their cytoskeleton into stress fibres, disrupt the tubular basement membrane, and migrate into the interstitium, where they synthesize increasing amounts of ECM, finally leading to renal fibrosis [114–116].

Many studies indicate that TGF-β is one of the most important profibrotic regulators of renal fibrosis in progressive CKD and stimulates the accumulation of matrix proteins to induce ECM, inhibits matrix degradation, and regulates myofibroblast activation [117–120]. Based on the role of TGF-β, many therapeutic approaches involving the inhibition of TGF-β have been tested in experimental models of CKD and clinical trials, such as the administration of neutralizing anti-TGF-β antibodies and small interfering RNAs that target the TGF-β type II receptor, which can reduce structural renal injury and decrease renal fibrosis in CKD [121–123]. It has been reported that klotho inhibition increases TGF-β1 expression in mice with renal fibrosis that has been induced by unilateral ureteral obstruction (UUO), and TGF-β1 reduces klotho expression in renal cultured epithelial cells, suggesting that decreased klotho expression enhances TGF-β1 activity and that klotho deficiency is not only a cause but also a result of renal fibrosis in CKD [124]. In contrast, soluble klotho protein directly binds to the TGF-β type-II receptor and inhibits TGF-β1 binding to cell surface receptors, thereby inhibiting TGF-β1 signalling in mice with UUO-induced renal fibrosis. Moreover, klotho decreases epithelial marker expression and increases mesenchymal marker expression to suppress the TGF-β1-induced epithelial-to-mesenchymal transition in renal epithelial cells [125]. These results indicate that klotho can suppress renal fibrosis by inhibiting TGF-β1 activity.

Another principal profibrotic molecule is named angiotensin II (Ang II); this molecule modulates fibrosis by direct effects on the matrix and by up-regulating the expression of other factors, such as TGF-β [126, 127], connective tissue growth factor [128], plasminogen activator inhibitor-1 [129], tumour necrosis factor-α, and NF-ĸB [113, 130]. Furthermore, data have shown that Ang II-induced renal damage suppresses klotho expression, whereas the induction of klotho gene expression mitigates Ang II-induced renal damage [131]. In addition, soluble klotho has been shown to inhibit Wnt and IGF-1 signalling, which can promote the epithelial-to-mesenchymal transition and myofibroblast activation [125, 132]. Recent studies also show that exogenous klotho decreases high glucose-induced fibronectin and cell hypertrophy via the ERK1/2-p38 kinase signalling pathway to attenuate diabetic nephropathy in vitro [133] and that the administration of klotho protein suppresses renal tubulo-interstitial fibrosis and UUO-induced renal fibrosis, at least partly, by controlling basic fibroblast growth factor-2 signalling in vivo [134]. These results raise the possibility that soluble klotho may function as a renal-protective factor against fibrosis by inhibiting multiple signalling pathways.

### Potential use of klotho in human chronic kidney disease

#### A potential biomarker for CKD

CKD is not easy to detected at early stage of CKD and thus it is very difficult to make an early and accurate diagnosis. And there are no biomarkers which are able to be measured easily, sensitively, reliably, and specially, in correlation with presence, development, and complications of CKD [135]. As described previously, renal klotho deficiency is highly associated with ion disorders, VC, inflammation, renal fibrosis, and mineral bone disorder, which are all characteristics of CKD. It has been shown that soluble klotho in the circulation starts to decline early in stage 2 CKD and urinary klotho possibly declines even earlier 1 [14]. In addition, data show that klotho deficiency in CKD can enhance the renal tubular and vascular cell senescence induced by oxidative stress [136, 137] and can result in defective endothelial function and impaired vasculogenesis [138]. Together, these findings indicate that klotho deficiency is closely correlated with the development and progression of CKD and extrarenal complications. Thus, soluble klotho deficiency seems to have diagnostic potential, serving as an early and sensitive biomarker of CKD.

Many researchers have investigated the possibility of using klotho as a biomarker for CKD. CKD-MBD is one of the striking features associated with the high morbidity and mortality of cardiovascular events in CKD and ESRD [51, 139]. Abnormal mineral metabolism includes high serum phosphate, FGF23, and PTH levels, which are closely associated with or even induced by klotho deficiency [14, 140–142]. Clinical studies in patients with CKD have shown that soluble klotho is lower than normal (519 ± 183 versus 845 ± 330 pg/mL, *P* < .0001) in renal patients, and soluble klotho is positively correlated with serum calcium and negatively correlated with serum phosphate, PTH, and FGF23, suggesting that soluble klotho might reflect the ensuing tubular resistance to FGF23, which could be an early marker of CKD-MBD [143, 144]. Recently, another clinical study suggested that soluble klotho is significantly associated with phosphate reabsorption independently of FGF-23, which may be a marker of phosphate reabsorption [145]. Therefore, soluble klotho seems to be a marker for disorders of phosphate and bone metabolism in CKD.

GFR, the gold standard for assessing kidney function, is significantly decreased in CKD [112]. Clinical and experimental studies have shown that this significant decrease in klotho in the kidneys is positively associated with estimated GFR (eGFR) in CKD samples [144–147]. Several other studies have confirmed the positive correlation between klotho levels (in serum and urine) and eGFR in adult patients with CKD [7, 33]. Moreover, both serum and urine klotho levels are independently associated with eGFR in patients with CKD [33, 148]. Another study showed that serum klotho levels are progressively lower with advancing CKD stage, with an adjusted mean decrease of 3.2 pg/mL for each 1 mL/min/1.73 m^2^ eGFR decrease [149]. Consistently, a similar positive correlation between plasma klotho levels and eGFR was shown in children with CKD [150]. These results suggest that the decrease in soluble klotho may mirror an eGFR decrease in patients with CKD.

However, some researchers obtained adverse results. Sarah Seiler et al. analysed a large cohort of 312 patients with stage 2-4 CKD and found that plasma klotho levels were not significantly associated with eGFR or other calcium-phosphate metabolism parameters in these patients [151]. Similarly, in a prospective observational study among 444 patients with CKD stages 2-4, klotho levels were not significantly related to cardiovascular outcomes [152]. These results indicate that plasma klotho levels are not related to kidney function and do not predict adverse outcome in patients with CKD. There may be two reasons for this contradictory data. One is age. Yamazaki et al. suggested that soluble klotho levels are correlated with age, finding that klotho levels are higher in children (mean age 7.1±4.8 years) than in adults [153]. Shimamura et al. also reported significantly lower klotho levels in CKD stage 2-5 patients than in CKD stage 1 patients. Moreover, this finding was largely based on data from four young individuals with normal eGFR and extremely high klotho levels, whereas klotho levels in the remaining participants did not predict adverse outcome of CKD [143, 151]. Furthermore, a recent clinical study found that an allele of the G-395A klotho gene polymorphism has a significantly higher frequency among children with CKD, suggesting that this mutant allele of klotho can be used as a risk marker for the development of ESRD and as a predictor of CVD in children [154]. Another reason may be the differences in sample size. The results obtained from some studies with small cohorts of CKD patients [155–157] were different from those obtained with a large cohort [151]. The idea of a decline in klotho levels with impaired kidney function has been further disputed by smaller studies [151, 155, 158].

Although the results of relations between circulating klotho levels and outcomes of CKD are contradictory, three commonly used commercial immunoassay products for measuring soluble klotho-- are available from IBL (IBL International GmbH, Hamburg, Germany), Cusabio (Cusabio Biotech, Wuhan, China), and USCN (USCN Life Science Inc., Wuhan, China) [159]. Only the IBL kit provides information on epitope specificity [159]. However, researchers have found that these assays exhibited poor performance, including a lack of unit standardization in readouts, and the assays have to be improved to produce accurate results before they can provide reliable conclusions [160].

#### As a potential treatment strategy for CKD

Although the causes of CKD are multifactorial, klotho deficiency is significantly associated with the development and progression of CKD and extrarenal complications. Many clinical and animal studies have suggested that when the klotho-deficient state in CKD is rescued, the renal function, morphologic lesion, and complications of CKD are obviously improved [4, 14, 16, 135, 148, 161]. For example, the administration of soluble klotho protein significantly attenuated UUO-induced renal fibrosis and suppressed the expression of fibrosis markers and TGF-β1 target genes, such as *Snail* and *Twist* [125]. Furthermore, klotho connected intermedin 1-53 to the suppression of VC in CKD rats [162], and klotho supplementation suppressed the renin-angiotensin system to ameliorate Adriamycin nephropathy. In addition, klotho protein appeared to suppress the epithelial-mesenchymal transition by inhibiting TGF-β and Wnt signalling [163]. Therefore, klotho deficiency may not only be a pathogenic intermediate in the acceleration of CKD progression but may also be a major contributor to chronic complications, such as CKD-MBD and cardiovascular diseases in CKD. Conceivably, any therapy that restores the klotho level by supplementation with exogenous klotho and/or the up-regulation of endogenous klotho production might be a novel treatment strategy for CKD [14].

Several methods are dependent on various mechanisms to increase klotho expression (Table 1) [14]; these includethe following: (1) Demethylation. Methylation of the klotho gene promoter reduces its activity by 30% to 40%, whereas DNA demethylation increases klotho expression 1.5-fold to threefold [164]. (2) Deacetylation. Data show that the TNF and TWEAK-induced down-regulation of klotho expression in the kidney and kidney cell lines can be blunted by the inhibition of histone deacetylase [74]. (3) Drugs. Several drugs on the market have been shown to up-regulate klotho expression in vivo and/or in vitro, including PPAR-γ agonists [165], angiotensin II-type I receptor antagonists [166], vitamin D active derivatives [167, 168], and intermedin [98]. (4) Klotho gene delivery. Klotho gene delivery through a viral carrier has been shown to effectively improve multiple pathophysiological phenotypes in klotho-deficient mice [169], thereby preventing the progression of kidney damage in rat models [170] and improving VC and endothelial function in CKD [80]. (5) Administration of soluble klotho protein. Increasing circulating klotho levels through the administration of soluble klotho protein, which is the cleaved, full-length extracellular domain of membrane klotho, is more direct, safer, and an easier modality to restore endocrine klotho deficiency [14, 72]. Animal studies have shown that the bolus administration of soluble klotho protein is a safe and effective means for protecting against kidney injury and preserving renal function [14, 72].

**Table 1:** Potential treatment strategies for CKD via the up-regulation of klotho

Methods	Mechanism
DNA demethylation	Methylation of the klotho gene promoter reduces its activity by 30-40%
Histone deacetylation	Hyperacetylation of histone in the klotho promoter down-regulates klotho expression
Drugs: PPAR-γ agonists, angiotensin II-type I receptor antagonists, statin, vitamin D active derivatives, intermedin	These drugs can up-regulate klotho expression in vitro and in vivo
Delivery of klotho cDNA	The klotho gene is transfected by viral carrier into target cells or animal models
Soluble klotho protein administration	Recombinant klotho protein, which is the cleaved, full-length extracellular domain of membrane klotho, can be injected

PPAR-γ: proliferator-activated receptor-gamma; statin: 3-hydroxy-3-methylglutaryl CoA (HMG-CoA) reductase inhibitors

## Conclusions

As the FGF23 co-receptor, klotho mediates FGF23 to regulate mineral ion (such as calcium and phosphate) homeostasis via klotho-FGFR complexes. Moreover, a recent study acknowledged that klotho is an on-demand non-enzymatic molecular scaffold protein that promotes FGF23 signalling. The identification of lipid rafts and sialogangliosides as the membrane receptors of soluble klotho helps us to understand more about how klotho functions as a circulating hormone or local autocrine/paracrine factor. However, klotho functions exert pleiotropic actions in the circulation. Thus, the klotho crystal structure, secretion, and regulation mechanism should be clarified in detail. A further understanding of the relation between klotho levels and CKD as well as its potential applications in vivo is very important for future therapeutic application.

## Abbreviations

1,25(OH)2D: 1α,25-dihydroxyvitamin D3
AKI: Acute kidney injury
Ang II: Angiotensin II
CKD: Chronic kidney disease
ECM: Extracellular matrix
ERK: Extracellular signal-regulated kinase
ESRD: End-stage renal disease
FGF: Fibroblast growth factor
FGFR: FGF receptors
GFR: Glomerular filtration rate
ICAM-1: Intercellular adhesion molecule 1
IL: Interleukin
IκB: Inhibitory κB
MBD: Mineral bone disorder
NF-κB: Nuclear factor κB
NHERF: Na+/H+ exchange regulatory cofactor
Nox2: NAPDH oxidase 2
PTH: Parathyroid hormone
RIG-I: Acid-inducible gene-I
SGK: Serum/glucocorticoid-regulated kinase
SMCs: Smooth muscle cells
TGF-β: Transforming growth factor β
TNF: Tumour necrosis factor
TRPV5: Transient receptor potential vannilloid-5
TWEAK: TNF-related weak inducer of apoptosis
UUO: Unilateral ureteral obstruction
VC: Vascular calcification
VCAM-1: Vascular cell adhesion protein 1
WNK4: With-no-lysine kinase 4
